# Dilated Cardiomyopathy in Children: A Diverse Etiological Profile

**DOI:** 10.7759/cureus.108972

**Published:** 2026-05-16

**Authors:** Soukayna Setouani, Asmae Mehdaoui, Nidale Hazzab, Samira Eddaoudi, Kawtar Khabbach, Yousra El Boussaadni, Abdallah Oulmaati

**Affiliations:** 1 Pediatrics, Centre Hospitalier Universitaire Mohammed VI de Tanger, Tangier, MAR

**Keywords:** aortic coarctation, dilated cardiomyopathy, echocardiography, etiology, genetic cardiomyopathy, heart failure, l-carnitine deficiency, metabolic disorders, myocarditis, pediatrics

## Abstract

Introduction

Dilated cardiomyopathy (DCM) is the most common form of cardiomyopathy in children and represents a major cause of heart failure and mortality. Its etiological diagnosis often remains complex, particularly in resource-limited settings.

Methods

This was a retrospective descriptive study conducted over a 26‑month period (February 2024 to April 2026) in the pediatric department of the University Hospital of Tangier, Morocco. All children diagnosed with dilated cardiomyopathy were included. Clinical, etiological, and outcome data were analyzed.

Results

Among 40 cases of cardiomyopathy, 20 (50%) were dilated cardiomyopathy. The mean age was four years (range: two months to 13 years), with a male predominance (sex ratio = 1.8). All patients were symptomatic at diagnosis. The mean ejection fraction was 34.6%. Mitral regurgitation was observed in 70% of cases, and intracavitary thrombus in 5%. Identified etiologies included myocarditis (15%), genetic causes (15%), aortic coarctation (15%), chronic renal failure (10%), rickets-related hypocalcemia (10%), coronary anomalies (10%), and L‑carnitine deficiency (5%). No etiology was identified in 15% of cases. All patients received heart failure treatment. Mortality was 20%, while 65% of patients showed improvement in cardiac function during follow‑up.

Conclusion

Dilated cardiomyopathy in children remains a serious condition with multiple etiologies and a non‑negligible mortality rate. Early etiological investigation is essential to guide management and improve prognosis.

## Introduction

Dilated cardiomyopathy (DCM) is characterized by ventricular chamber enlargement and systolic dysfunction in the absence of abnormal loading conditions or coronary artery disease sufficient to explain the myocardial impairment [1,2]. It results from direct involvement of the cardiac muscle and may affect the left ventricle alone or both ventricles [3]. Cardiomyopathies are currently defined and managed according to contemporary international recommendations, particularly the 2023 European Society of Cardiology guidelines, which emphasize a phenotype-based approach integrating clinical evaluation, multimodality imaging, genetic assessment, and family screening [4]. Contemporary classifications categorize cardiomyopathies into dilated, hypertrophic, restrictive, arrhythmogenic, and unclassified forms according to their morphological and functional characteristics [4,5].

According to the American Heart Association (2006), cardiomyopathies may be hereditary, acquired, or mixed. DCM is classified as a “mixed” cardiomyopathy because of its broad and heterogeneous etiological spectrum [5]. The European Society of Cardiology further distinguishes familial and non-familial forms of DCM [6]. Familial forms are mainly associated with monogenic disorders, including mutations affecting sarcomeric, cytoskeletal, or mitochondrial proteins. Non-familial forms may result from multiple acquired conditions, such as infectious and inflammatory diseases (particularly viral myocarditis), metabolic disorders including L-carnitine deficiency and fatty acid oxidation defects, nutritional deficiencies such as hypocalcemia, toxic exposures, endocrine disorders, systemic diseases, or structural cardiovascular abnormalities. In some patients, despite extensive investigations, no underlying cause can be identified, and the condition is therefore classified as idiopathic cardiomyopathy [6].

DCM represents the most common form of cardiomyopathy in children, accounting for nearly 50-60% of pediatric cardiomyopathies [7,8]. Although uncommon, its incidence in the pediatric population remains significant because of its association with severe heart failure, arrhythmias, thromboembolic complications, and sudden cardiac death [8-10]. The reported incidence is estimated at less than 2 cases per 100,000 children annually, with a prevalence of approximately 2.6 per 100,000 children [8-12]. Several studies have reported a male predominance [8,13], although sex distribution may vary across populations [14,15]. In addition, racial and geographic disparities have been described, with a higher incidence reported among Black children compared to White children [8,16].

Pediatric DCM is one of the leading causes of chronic heart failure and remains the most common indication for heart transplantation in children older than one year [7]. Clinically, pediatric DCM most frequently presents with signs and symptoms of heart failure, including dyspnea, tachypnea, feeding difficulties in infants, fatigue, exercise intolerance, respiratory distress, and peripheral edema. In severe cases, arrhythmias, thromboembolic events, or cardiogenic shock may occur. Diagnosis relies primarily on non-invasive investigations, particularly echocardiography, which remains the cornerstone for confirming ventricular dilatation, assessing systolic dysfunction, and monitoring disease progression. Despite advances in diagnostic and therapeutic strategies, the prognosis of pediatric DCM remains poor, with mortality rates still ranging from 10% to 50% depending on the underlying etiology and disease severity [8,17,18].

The aim of this study was to describe the experience of the Pediatric Department of Mohammed VI University Hospital in Tangier and to analyze the epidemiological, clinical, etiological, therapeutic, and prognostic characteristics of pediatric dilated cardiomyopathy in our setting. Particular attention was given to distinguishing potentially reversible etiologies from idiopathic forms, as early identification of treatable causes may significantly improve outcomes in resource-limited settings.

## Materials and methods

This single-center retrospective descriptive observational study was conducted in the Department of Pediatrics of Mohammed VI University Hospital in Tangier, Morocco, over a 26-month period extending from February 2024 to April 2026. The study aimed to analyze the epidemiological, clinical, etiological, therapeutic, and prognostic characteristics of pediatric DCM in our regional setting, with particular emphasis on identifying potentially reversible etiologies. The study was conducted in accordance with the principles of the Declaration of Helsinki. Patient confidentiality and anonymity were strictly respected throughout the study. Data were anonymized prior to analysis. Due to the retrospective observational design of the study, informed consent was waived.

Study population

All consecutive newborns, infants, children, and adolescents aged 0-15 years diagnosed with DCM during the study period were included. The diagnosis of DCM was established by transthoracic echocardiography based on the presence of left ventricular or biventricular dilatation associated with systolic dysfunction, in the absence of abnormal loading conditions or congenital heart disease sufficient to explain the myocardial impairment. Patients presenting with congenital heart disease responsible for ventricular dysfunction, primary valvular disease, severe systemic hypertension, or secondary cardiomyopathy related to volume or pressure overload were excluded from the study.

Sample size justification

Due to the retrospective nature of the study and the rarity of pediatric DCM, no formal sample size calculation was performed. Instead, all consecutive eligible patients admitted to the Department of Pediatrics during the study period were included. This exhaustive recruitment strategy was intended to provide a representative overview of the local experience with pediatric DCM and to allow a comprehensive assessment of its clinical and etiological spectrum.

Data collection

Clinical, biological, radiological, and echocardiographic data were retrospectively collected from electronic medical records and echocardiography reports using a standardized data collection form. Demographic variables included age, sex, consanguinity, and family history of cardiomyopathy or sudden cardiac death. Baseline clinical characteristics included symptoms at presentation, signs of heart failure, hemodynamic status, and associated extracardiac manifestations. All patients underwent electrocardiography, chest radiography, transthoracic echocardiography, and standard biological investigations. Electrocardiographic analysis focused on rhythm disturbances, conduction abnormalities, and signs suggestive of myocardial ischemia. Chest radiography was evaluated for cardiomegaly and pulmonary vascular congestion. Echocardiographic assessment included ventricular chamber dimensions, left ventricular systolic function, valvular abnormalities, intracardiac thrombus, and associated structural abnormalities. Etiological investigations were performed according to the clinical context and included assessment for myocarditis, metabolic disorders, nutritional deficiencies, systemic diseases, genetic causes, coronary anomalies, and idiopathic forms when no etiology could be identified. Additional investigations, including endocrine assessment and metabolic testing, were performed when clinically indicated. Treatment modalities included conventional heart failure therapy, supportive care, anticoagulation when necessary, and etiology-specific management whenever a reversible or treatable cause was identified. Clinical outcomes during follow-up included mortality, improvement or deterioration of cardiac function, persistence of symptoms, and rehospitalization.

Statistical analysis

Data were entered and analyzed using Microsoft Excel 2016 (Microsoft Corporation, Redmond, Washington, United States) and IBM SPSS Statistics for Windows, version 28 (IBM Corp., Armonk, New York, United States). Quantitative variables were expressed as mean ± standard deviation (SD), median, and range according to data distribution, whereas qualitative variables were presented as absolute numbers and percentages. Baseline demographic, clinical, biological, echocardiographic, etiological, and outcome characteristics were summarized using descriptive statistics. Comparative analyses were performed to explore potential associations between etiological profiles, severity of cardiac dysfunction, and clinical outcomes. Categorical variables were compared using the Chi-square test or Fisher’s exact test when appropriate, while quantitative variables were analyzed using Student’s t-test or Mann-Whitney U test according to distribution normality. A p-value <0.05 was considered statistically significant. However, given the relatively limited sample size, inferential analyses were considered exploratory and interpreted with caution.

## Results

Epidemiological characteristics

**Figure 1:**
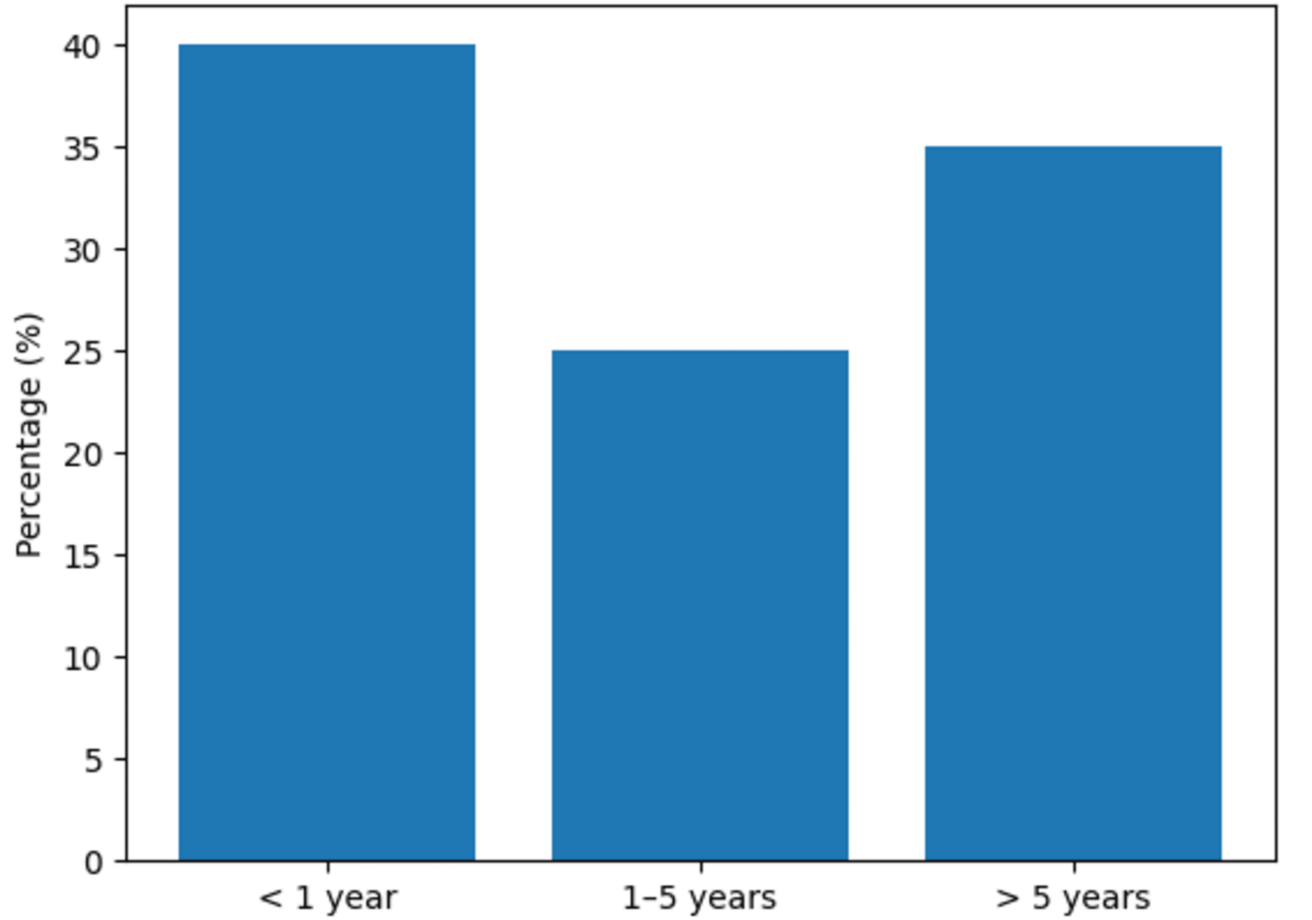
Age distribution of patients with dilated cardiomyopathy.

During the 26-month study period, 40 cases of cardiomyopathy were identified in our department, among which 20 patients presented with dilated cardiomyopathy, representing 50% of all cardiomyopathies diagnosed during the study period. The age at diagnosis ranged from two months to 13 years, with a mean age of four years. Infants younger than one year represented the largest subgroup, accounting for 40% (n=8) of cases, followed by children older than five years (35%, n=7) and those aged between one and five years (25%, n=5). The age distribution of the study population is illustrated in Figure 1.

**Figure 2:**
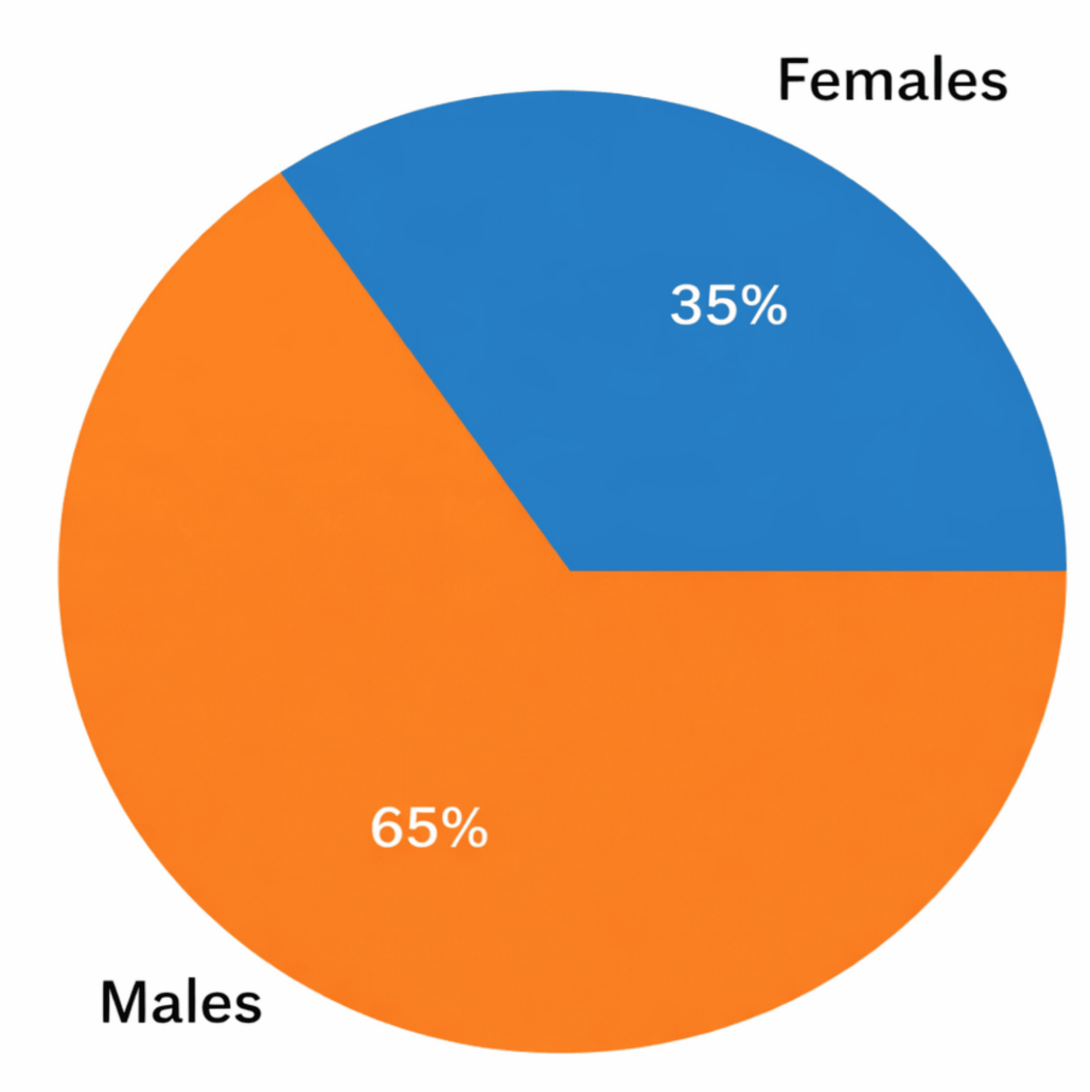
Sex distribution of the study population.

A clear male predominance was observed, with 13 male (65%) and seven female (35%) patients, corresponding to a male-to-female ratio of 1.8 (Figure 2). In addition, parental consanguinity was identified in 15% (n=3) of patients, while a family history of cardiomyopathy or sudden cardiac death was reported in 10% (n=2) of cases, supporting the possible contribution of genetic factors in pediatric DCM.

Clinical presentation

**Figure 3:**
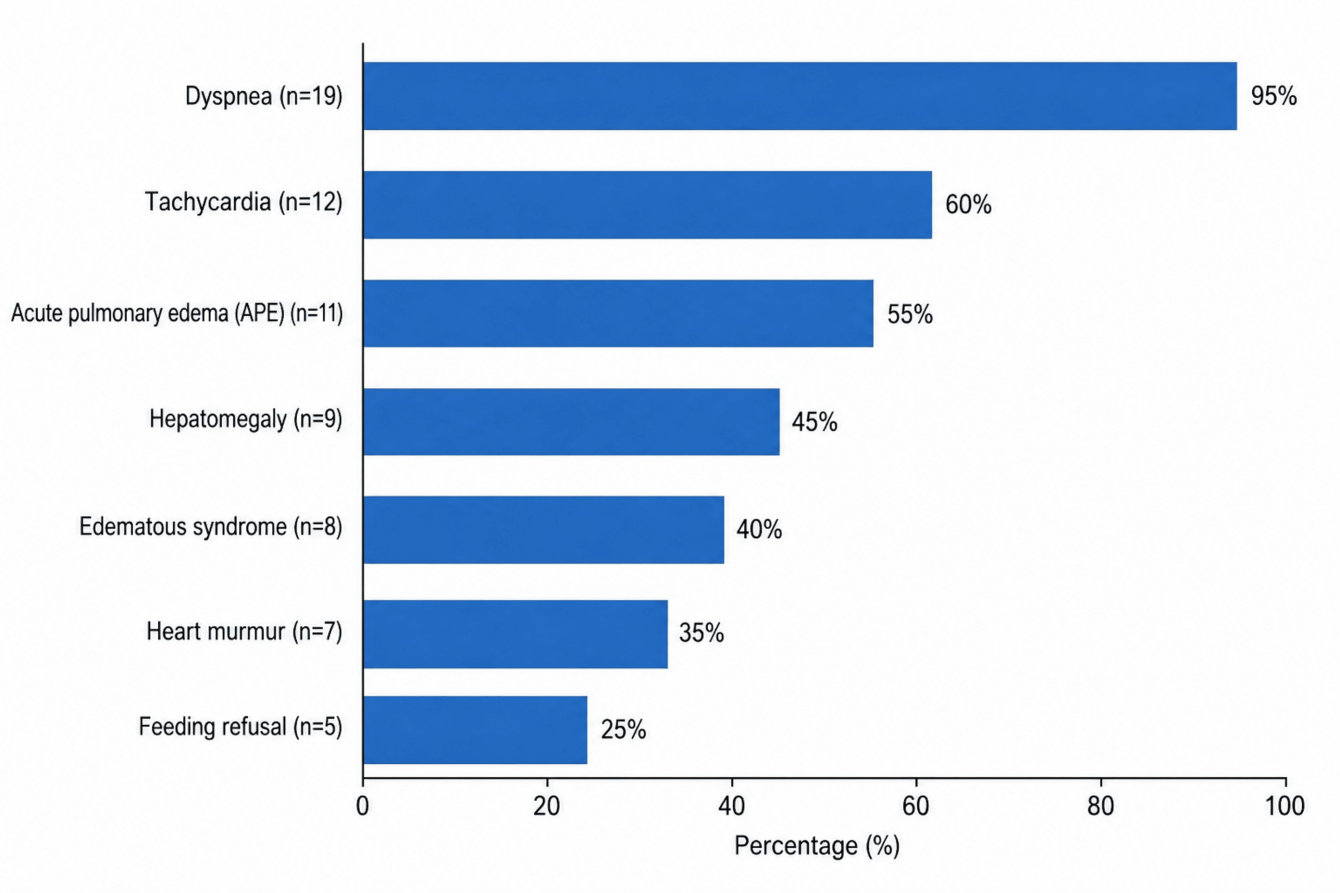
Clinical manifestations at diagnosis in children with dilated cardiomyopathy.

All patients were symptomatic at the time of diagnosis, with heart failure representing the predominant clinical presentation. Dyspnea was the most frequent symptom, affecting 95% (n=19) of patients, and was particularly severe in older children, most of whom presented with New York Heart Association (NYHA) class III or IV symptoms. Clinical presentation varied according to age. In infants, respiratory distress was frequently associated with feeding difficulties or refusal to breastfeed (n=6, 30%), whereas older children more commonly exhibited persistent dyspnea at rest (n=7, 35%), exercise intolerance (n=5, 25%), and peripheral edema (n=4, 20%). Signs of systemic fluid overload were observed in 55% (n=11) of patients. The onset of symptoms was progressive in most cases; however, acute and severe presentations were also observed. Four patients (20%) presented with cardiogenic shock at admission, requiring urgent medical management. The spectrum of presenting manifestations is summarized in Figure 3.

Paraclinical data

**Figure 4:**
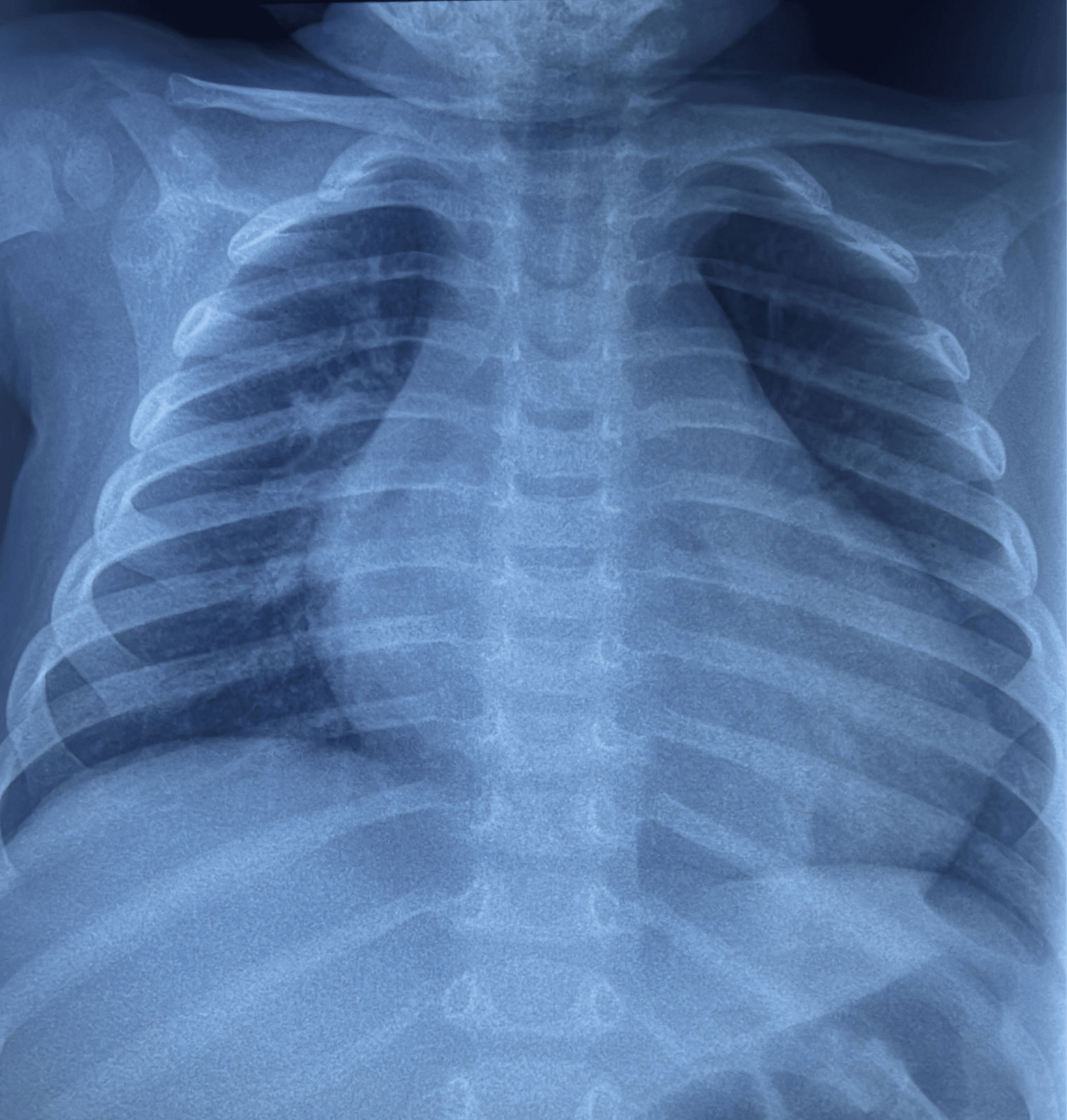
Cardiomegaly in a child with dilated cardiomyopathy.

Electrocardiographic abnormalities were observed in all patients and mainly included rhythm disturbances and signs suggestive of myocardial ischemia. Pathological Q waves in leads I and aVL raised suspicion of anomalous origin of the left coronary artery in some patients. Tachyarrhythmias were also identified and were considered either secondary to myocardial dysfunction or potentially involved in tachycardia-induced cardiomyopathy. Radiological assessment systematically demonstrated cardiomegaly, with a mean cardiothoracic index of 0.64. Pulmonary vascular congestion was present in more than half of the patients, reflecting the advanced stage of heart failure at diagnosis. A chest radiograph of a study participant demonstrating marked cardiomegaly is shown in Figure 4.

Biological investigations revealed renal dysfunction and hepatic cytolysis in a substantial proportion of patients, likely reflecting systemic consequences of severe heart failure and low cardiac output. Etiological investigations identified potentially treatable metabolic causes, including L-carnitine deficiency, emphasizing the importance of systematic metabolic evaluation in pediatric DCM.

Transthoracic Doppler echocardiography was the cornerstone investigation for diagnosis and follow-up. All patients exhibited ventricular dilatation associated with impaired systolic function. The mean left ventricular ejection fraction was markedly reduced at 34.6%, reflecting advanced myocardial dysfunction in most patients. Valvular involvement was frequent, particularly mitral regurgitation, which was identified in 70% of patients and was predominantly mild to moderate in severity. Tricuspid regurgitation was less common. Intracavitary thrombus was identified in one patient, highlighting the thromboembolic risk associated with severe ventricular dysfunction. Detailed echocardiographic findings are summarized in Table 1. 

**Table 1:** Echocardiographic characteristics.

Echocardiographic parameter	Number of patients	Percentage
Mean ejection fraction		34.6%
Mitral regurgitation : Absent	6	30%
Grade I	8	40%
Grade II	5	25%
Grade III	1	5%
Tricuspid regurgitation : Absent	15	75%
Grade I	4	20%
Grade II	1	5%
Grade III	0	0%
Intracavitary thrombus	1	5%

**Figure 5:**
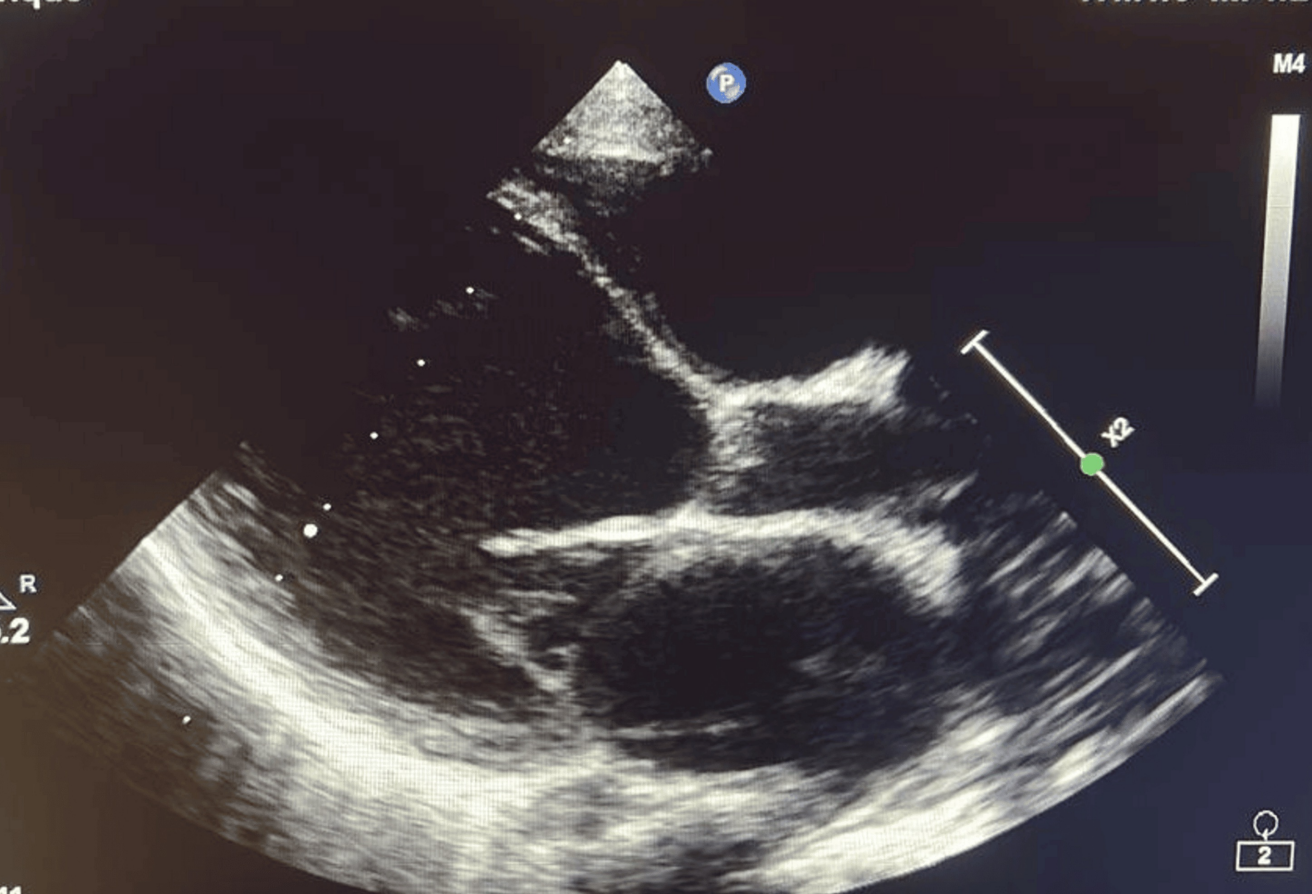
Dilated cardiomyopathy.

**Figure 6:**
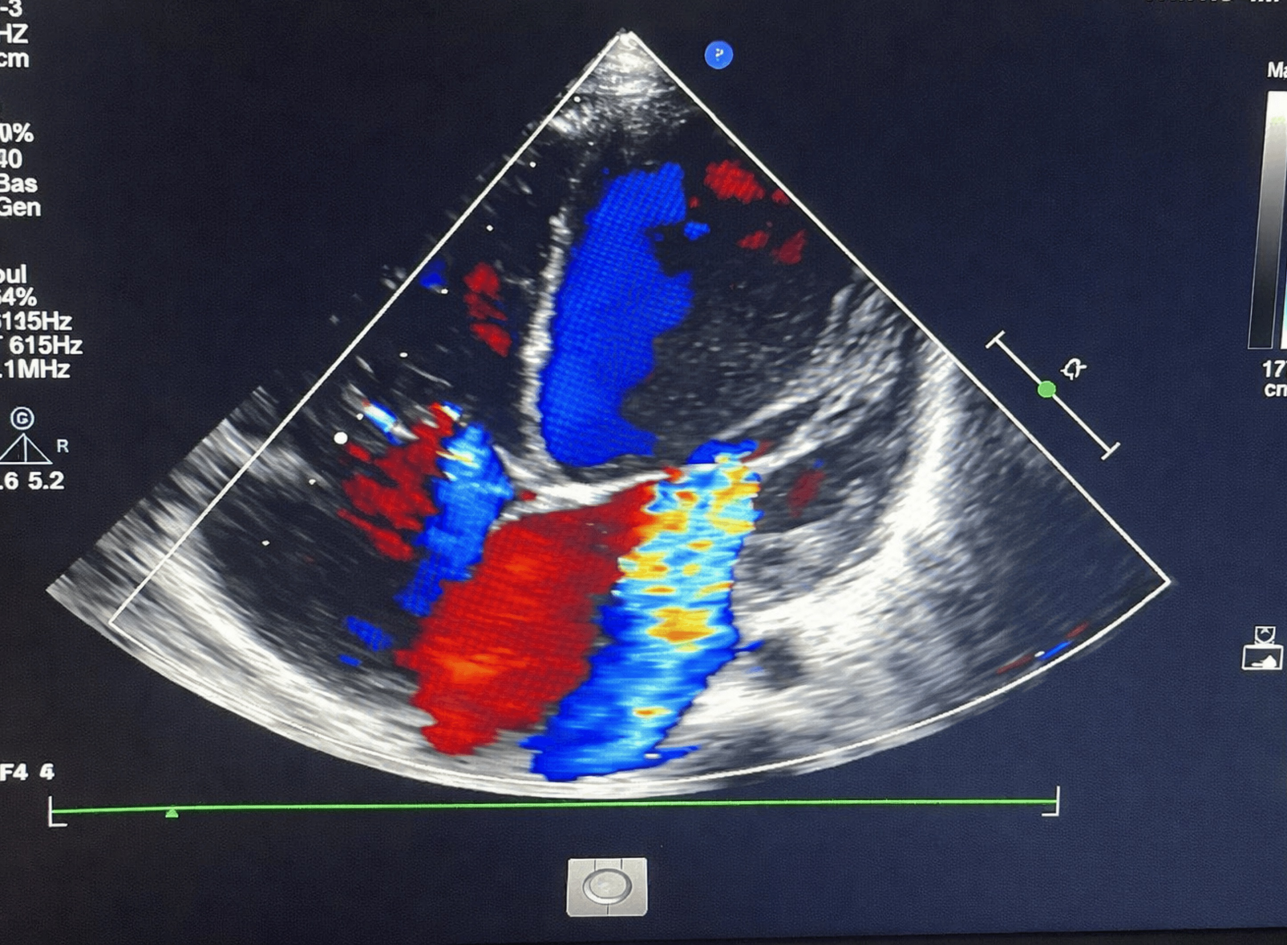
Mitral regurgitation.

Representative echocardiographic images of study participants, illustrating ventricular dilatation and mitral regurgitation, are presented in Figures 5, 6. 

Etiological profile

Etiological assessment revealed marked heterogeneity, reflecting the multifactorial nature of pediatric DCM. Myocarditis, genetic causes, and aortic coarctation each accounted for 15% of cases, making them the most frequently identified etiologies in our cohort. Potentially reversible causes, including hypocalcemia secondary to rickets, metabolic disorders, and L-carnitine deficiency, were also identified, underlining the importance of comprehensive etiological investigations in children presenting with DCM. In contrast, idiopathic forms accounted for 15% of cases despite extensive investigations. The etiological spectrum observed in our cohort is summarized in Table 2.

**Table 2:** Etiological distribution.

Cause	Frequency (Percentage)
Aortic coarctation	3 (15%)
Chronic renal failure	2 (10%)
Hypocalcemia related to rickets	2 (10%)
Myocarditis	3 (15%)
L‑carnitine deficiency	1 (5%)
Medium‑chain acyl‑CoA dehydrogenase (MCAD) deficiency	1 (5%)
Chronic anemia due to familial lymphohistiocytosis	1 (5%)
Congenital coronary artery anomaly	2 (10%)
Undetermined cause	3 (15%)
Genetic cause	2 (10%)

Treatment

All patients received heart failure treatment adapted to their clinical condition, mainly combining diuretics, angiotensin-converting enzyme inhibitors, and digoxin when indicated. An antiplatelet agent was prescribed in patients with a left ventricular ejection fraction below 30%, and curative anticoagulation was initiated in cases of intracavitary thrombus. Etiology-specific treatment was introduced whenever an underlying cause was identified.

Outcome during follow‑up

**Figure 7:**
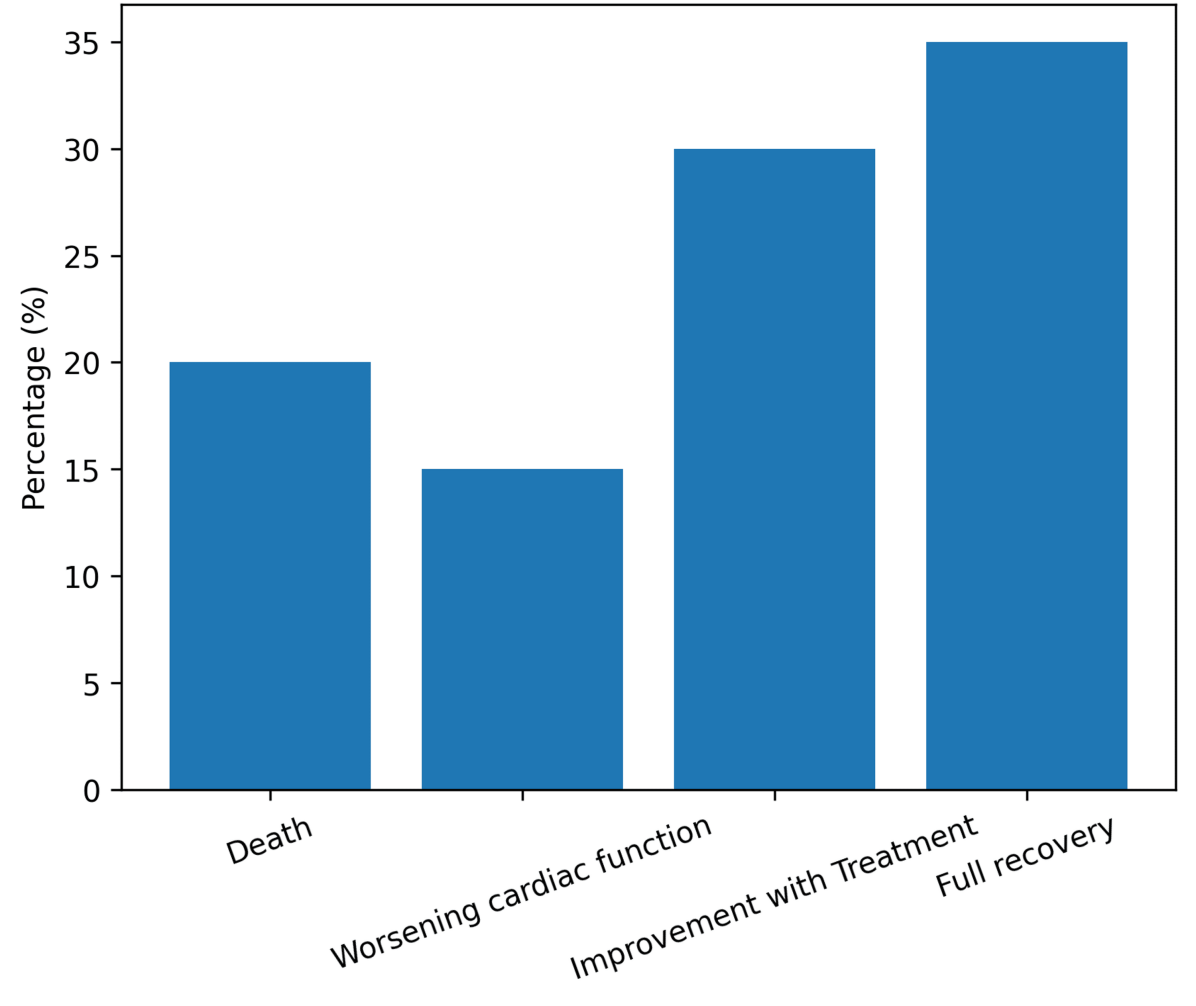
Clinical outcomes during follow-up.

Clinical evolution during follow-up was heterogeneous. Although a significant proportion of patients demonstrated partial or complete recovery under treatment, mortality remained substantial at 20% (n=4), confirming the severity of pediatric DCM. Complete recovery of ventricular function was achieved in 30% (n=6) of patients, whereas 15% (n=3) experienced progressive deterioration despite medical management. These findings highlight the variable prognosis of pediatric DCM, which largely depends on the underlying etiology, severity of myocardial dysfunction at diagnosis, and response to treatment. Clinical outcomes observed during follow-up are summarized in Figure 7.

## Discussion

DCM is the most common form of cardiomyopathy in children and represents a major cause of pediatric heart failure. However, its true incidence remains difficult to determine and is likely underestimated. Epidemiological studies conducted in Australia [12], Finland [17], and North America [10] report an incidence ranging from 0.75 to 1.24 cases per 100,000 children.

In our series, DCM accounted for 50% of cardiomyopathies, a finding consistent with the literature, which reports similar proportions (51% in North America, 58.6% in Australia, and 52% in Finland). The mean age at diagnosis in our study was 48 months, higher than that reported in several international series: 7.5 months in Australia [12], 13 months in Finland [17], and 21.6 months in North America [10]. This discrepancy may be explained by differences in inclusion criteria, particularly the age limits of the studied populations. Nevertheless, DCM remains a disease of infants and young children, with a high proportion of early diagnoses: 40% of cases before one year of age in our series, compared with 65% in Australia [12], 51% in Finland [17], and 41% in North America [12].

A male predominance was observed, consistent with some reports in the literature. Parental consanguinity was found in 15% of our patients, a rate close to that reported in the Australian series (8.8%) [19], suggesting a possible contribution of genetic factors. Clinically, dyspnea was the predominant symptom, present in 95% of patients regardless of age. In infants, it was frequently associated with feeding difficulties, particularly refusal to breastfeed, observed in 30% of cases. In older children, the clinical presentation was mainly characterized by congestive heart failure with peripheral edema, reported in 55% of patients. The fact that all patients were symptomatic at diagnosis may reflect delayed diagnosis and management, a situation frequently encountered in resource-limited settings.

DCM is characterized by left ventricular or biventricular dilatation associated with systolic dysfunction, most often resulting in global heart failure. Clinical presentation may vary from progressive onset to acute severe complications, including acute pulmonary edema, arrhythmias, thromboembolic events, or cardiogenic shock, although incidental diagnosis may occasionally occur. In our series, 80% of patients presented with heart failure at diagnosis, a rate comparable to those reported in international studies, including 89.7% in an Australian series [12] and 71% in an American series [10].

Echocardiography remains the key diagnostic and follow-up tool, confirming ventricular dilatation and systolic dysfunction [20]. In our study, the mean ejection fraction was 34.6%, with associated mitral regurgitation in 70% of cases and intracavitary thrombus in 5%. Etiological analysis reveals marked heterogeneity. In our series, identified causes included myocarditis, genetic causes, congenital heart disease (including aortic coarctation), metabolic disorders (L-carnitine deficiency), electrolyte disturbances (hypocalcemia), and systemic diseases. Three cases remained without an identified etiology. These findings are consistent with the literature, where idiopathic forms remain predominant (up to 76%), followed by myocarditis (12%) and familial forms (7%) [13,17,10]. The relatively low proportion of idiopathic cases in our series may reflect an extensive diagnostic approach. Moreover, the identification of potentially reversible causes, such as hypocalcemia, highlights the importance of a comprehensive etiological workup [10,21]. Pediatric DCM remains a disease with a poor prognosis, often progressing to refractory heart failure and representing the leading indication for heart transplantation in children. Five-year survival ranges from 15% to 50% across different series [20]. In our study, mortality was 20%.

Prognosis of pediatric DCM depends on multiple clinical, etiological, and paraclinical factors

Age at diagnosis has been identified as an important prognostic factor in pediatric dilated cardiomyopathy. Several studies reported poorer outcomes among children diagnosed at an older age, whereas infancy has also been associated with severe disease presentation in some cohorts [10,15,17,18,20-25]. In our series, half of the deaths occurred in infants younger than one year, supporting the prognostic influence of age at diagnosis. Family history has also been associated with poorer outcomes in pediatric dilated cardiomyopathy and may reflect the contribution of underlying genetic forms of the disease [17,19,26]. In our cohort, two deceased patients had a positive family history of cardiomyopathy or sudden cardiac death. Etiology strongly influences prognosis. Myocarditis-related DCM is generally associated with a more favorable outcome and a higher probability of recovery of ventricular function [27]. In our series, 80% of children who recovered ventricular function reported a febrile episode preceding symptom onset. Ventricular arrhythmias are generally associated with more severe myocardial dysfunction and poorer prognosis in pediatric DCM [17].

Cardiomegaly associated with pulmonary vascular congestion on chest radiography may also reflect advanced heart failure and severe disease. In our study, three of the four deceased children had a cardiothoracic index greater than 0.65 associated with pulmonary congestion at admission. Echocardiographic parameters provide important prognostic information. Previous studies identified left atrial enlargement and severe ventricular dilatation as markers associated with adverse outcomes [11,28]. In our cohort, severe systolic dysfunction was associated with poorer clinical evolution. Left ventricular systolic dysfunction remains one of the strongest predictors of mortality in pediatric DCM. In our series, two deceased patients had an ejection fraction below 20%, and one patient developed intracavitary thrombus, reflecting advanced myocardial impairment and increased thromboembolic risk.

Limitations

This study has several limitations. First, the relatively small sample size may limit the generalizability of the findings. Second, the retrospective design may have introduced information bias due to incomplete medical records. Third, the single-center setting may reduce external validity. Finally, the absence of long-term follow-up limits the assessment of long-term outcomes. Future multicenter prospective studies are needed to confirm these findings.

## Conclusions

DCM in children is a serious condition characterized by marked etiological heterogeneity and a potentially unfavorable prognosis. Early diagnosis and comprehensive etiological assessment are essential to optimize management and improve clinical outcomes, particularly in resource-limited settings. Our study aimed to describe the epidemiological, clinical, etiological, therapeutic, and prognostic characteristics of pediatric DCM in our center. An etiology was identified in 80% of cases, with heart failure representing the most frequent clinical presentation. Potentially reversible causes were also identified, emphasizing the importance of systematic etiological investigations in pediatric patients presenting with DCM.

Although pediatric DCM remains a rare disease, its prognosis may be severe, especially in advanced forms. Further investigations, including genetic testing, metabolic evaluation, cardiac MRI, virological studies, and, in selected cases, endomyocardial biopsy, may contribute to a more accurate etiological diagnosis and improved therapeutic management. The establishment of DCM registries would help better estimate the incidence, etiological spectrum, and outcomes of pediatric DCM. Early family screening should also be encouraged, particularly in suspected familial forms, as it may facilitate earlier diagnosis and reduce unnecessary investigations.
